# Renal cell -like carcinoma of the nasal cavity: a case report and review of the literature

**DOI:** 10.1186/s13000-017-0660-1

**Published:** 2017-10-17

**Authors:** Zhenwei-Chen, Zhaoming-Wang, Hongqi-Shi, Qinwei-Liu

**Affiliations:** 1 Department of Pathology, Jinhua Municipal Central Hospital, 351 Mingyue Road, Jinhua, 321000 Zhejiang Province People’s Republic of China; 2 Department of Pathology, The First Affiliated Hospital of Zhejiang University, 79 Qingchun Road, Hangzhou, 310000 Zhejiang Province People’s Republic of China

**Keywords:** Sinonasal tract, Renal cell-like carcinoma, Renal clear cell carcinoma

## Abstract

**Background:**

Sinonasal renal cell-like carcinoma (SRCLC) is an extremely rare low malignant tumor arising in the sinonasal tract, with histological mimicry of renal cell carcinoma.

**Case presentation:**

We present a case of sinonasal renal cell-like carcinoma in a 63-year-old male patient. Computer tomography(CT) scanning revealed a soft tissue mass at the left nasal cavity and choana. Histologically, the predominant tumor architecture was follicular to glandular with intervening fibrous septa. The tumor cells were uniform cuboidal to polyhedral with abundant clear or eosinophilic cytoplasm. Immunohistochemically, the tumor cells were strongly positive for CK7, EMA, vimentin, SOX10, S-100, and focally positive for CA9. During 6 months of follow-up, there was no clinical or radiological evidence of recurrence or metastasis.

**Conclusion:**

SRCLC has microscopic features which overlap with tumors that contain clear cells. Thus, several other tumors must be considered in the differential diagnosis of a tumor of the sinonasal region with clear cells, especially metastatic renal clear cell carcinoma. SRCLC is an indolent tumor and none of the reported SRCLC patients had metastatic disease.

## Background

SRCLC is a rare, low-grade malignant tumor, that was first described in 2002 [1, 2]. Microscopically, SRCLC is uniformly composed of a varied histological appearance, with glandular and follicular areas in various proportions, nested, solid, or papillary patterns may also be seen. SRCLC is characterized by strong positive expression for CK7, EMA,SOX10, S-100 and rarely for vimentin and CA9. When a histologically clear cell tumor appears in the nasal tract, the possibility of metastatic disease should be considered, particularly renal clear cell carcinoma(RCC). In addition to metastatic RCC, other diagnostic considerations for clear cell tumors in the paranasal sinuses include squamous cell carcinoma with clear cell change, mucoepidermoid carcinoma, melanoma, salivary clear cell carcinoma,metastatic clear cell variant of follicular thyroid carcinoma and epithelial-myoepithelial carcinoma and. We present one more rare case of SRCLC and discuss its histological features and the differential diagnosis.

## Case presentation

**Fig. 1:**
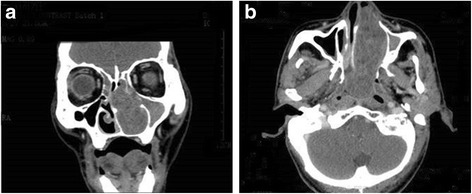
**a** A coronal CT image showing a soft tissue mass filling the left nasal cavity, causing obstruction of the left frontal and ethmoid sinuses. **b** Axial enhanced CT showed a soft tissue mass in the left nasal cavity featuring mild to moderate heterogeneous enhancement. The tumor extends posteriorly to the choana and nasopharyngeal region, with an indistinct border from the soft tissue of the lateral nasopharyngeal wall

**Fig. 2:**
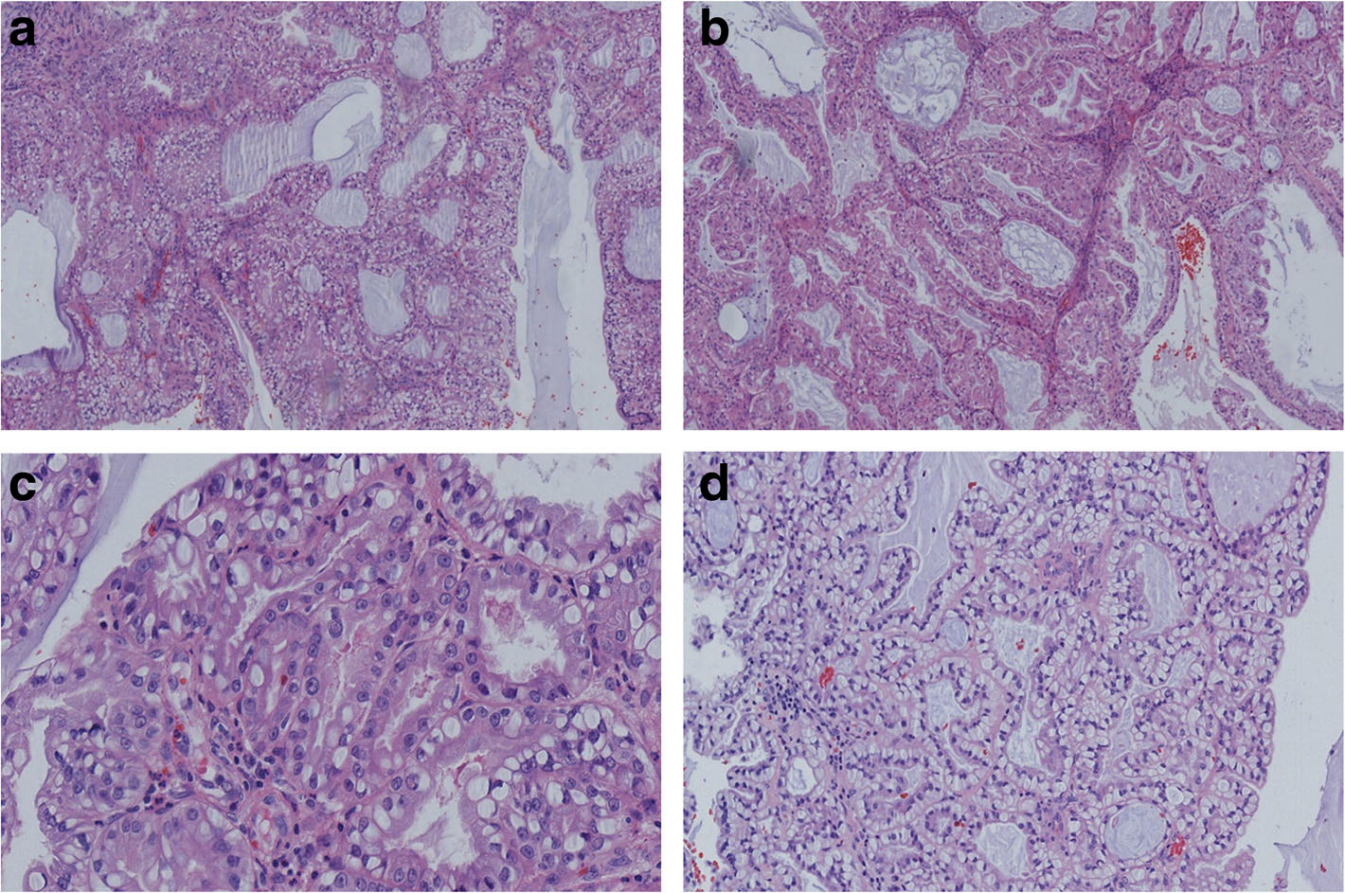
**a** The tumor consists of nests and follicles of polyhedral cells with abundant optically clear cytoplasm. Nuclear pleomorphism and mitotic activity are minimal. The follicles are filled with basophilic secretions(magnification × 100). **b** There is also micropapillary configuration that lacks fibrovascular cores(magnification × 100). **c** There are several intranuclear inclusions, which are quite unique to this tumor type (magnification × 400). **d** Some nuclei are in linear arrangement away from the basal aspects of the cells, which is a consistent feature in clear cell papillary renal cell carcinoma (magnification × 200)

**Fig. 3:**
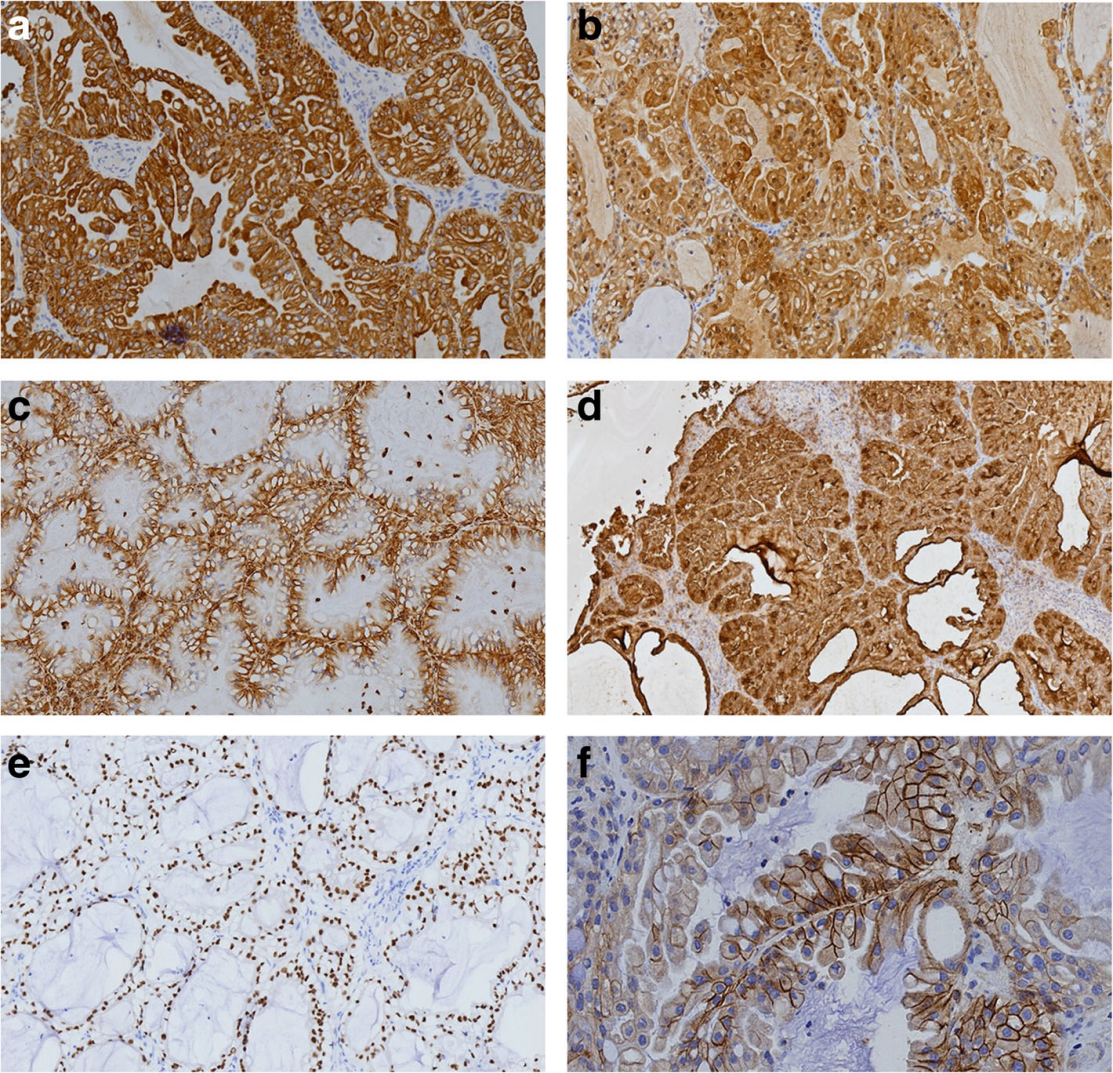
**a** Immunohistochemical examination showed positive expression of CK7(magnification × 100). **b** Immunohistochemical examination showed positive expression S-100(magnification × 100). **c** Immunohistochemical examination showed positive expression of Vimentin (magnification × 200).**d** Immunohistochemical examination showed positive expression of EMA(magnification × 100). **e** Immunohistochemical examination showed nuclear positive expression of SOX10(magnification × 200). **f** Immunohistochemical examination showed focally positive expression of CA9(magnification × 400)

A 63-year-old male presented to our otorhinolaryngology clinic with headache, left nasal obstruction and recurrent epistaxis. The patient complained of headache, dizziness and left nasal obstruction for 3 years. However, a recurrent epistaxis occurring three to four times a day for a week, was the reason he was referred to our hospital for medical treatment. The patient had no tumor history, and also no kidney tumor by general physical checkup. Nasal endoscopy examination showed a tumor completely occupying the left nasal cavity. CT scan of the paranasal sinuses was performed. The CT scan showed a soft tissue mass in the left nasal cavity and choana, with some bone compression but no invasive bone destruction (Fig. 1). A biopsy was performed and tissue was obtained in multiple pieces measuring in total 1.5X1X0.6 cm. Microscopically, the tumor consisted of glands and follicles, with focal micropapillary pattern, lined by cells with abundant clear or eosinophilic cytoplasm (Fig. 2a, b). The cells were uniform, cuboidal to polyhedral and the nuclei were slightly irregular to shrunken with coarse chromatin. Several intranuclear inclusions could be seen [Fig. 2c]. Some nuclei were in a linear arrangement away from the basal aspect of the cells, usually in the center of cytoplasm or more apical, with a classic feature of clear cell papillary renal cell carcinoma (Fig. 2d). No necrosis, mitotic activity, or perineural invasion was identified in the tumor. Immunohistochemically, the tumor cells showed strong cytoplasmic expression for CK7 (Fig. 3a), S-100 (Fig. 3b), vimentin (Fig. 3c), EMA (Fig. 3d), nuclear expression for SOX10 (Fig. 3e), focal membranous for CA9 (Fig. 3f), and were negative for thyroglobulin, actin, calponin, PAX8, CD10, CK20, CDX2, CD117, DOG-1 and SMA. Based on the histological and immunophenotypical findings, a diagnosis of sinonasal renal cell-like carcinoma was rendered. Later, surgery was performed under general anesthesia, followed by adjuvant radiation therapy. The patient is well with no disease progression at 6 months of follow up. The patient had a renal CT scan both at the time or following the initial diagnosis. No evidence of renal carcinoma was found.

## Discussion

Zur et al. first described a rare sinonasal tract carcinoma in 2002 as a histologic mimicker of renal clear cell carcinoma, which was named “sinonasal renal cell-like carcinoma” [1]. Additional reports have been identified, and there are currently 14 patients described in the English literature [1–9]. According to the review by BISHOP JA [6], the patients had a broad age distribution range from 22 to 75 years (mean 54). There was a female predominance of 9:4. The clinical manifestations, which are relatively nonspecific, include nasal obstruction, epistaxis, headache, epiphora and olfactory impairment. The most common site of involvement is the nasal cavity, followed by paranasal sinuses and nasopharynx [1, 3, 7–10].

Microscopically, SRCLC is composed of mixtures of follicular to glandular structures with intervening fibrous septa. The neoplastic cells are uniform, cuboidal to polyhedral with abundant clear to eosinophilic cytoplasm. There are slightly irregular to shrunken nuclei with coarse chromatin and a low nuclear: cytoplasmic ratio. The mitoses are inconspicuous and tumor necrosis, perineural invasion are absent [1–3, 6–9]. Some neoplastic cells contain intranuclear inclusions, which are a quite distinctive feature of this tumor [3, 7]. SRCLC is less vascular and pleomorphic than renal clear cell carcinoma. All the histological features described above are illustrated in our case.

Because of the presence of clear microscopic appearance, SRCLC have to be differentiated from many neoplasms. The top differential diagnosis is metastatic renal clear cell carcinoma(RCC), which not infrequently metastasizes to the nasal cavity [11, 12]. Strong staining for CK7, S-100 and SOX10 supports SRCLC and conversely, strong staining for PAX8 and CD10 supports metastatic RCC. In our case, the tumor cells were positive for CK7, S-100, SOX10 and negative for PAX-8 and CD10, arguing against a metastatic clear cell RCC. However, since CA9, EMA and vimentin are also expressed in both RCC and SRCLC [7, 13], they are of limited value in differentiating the two tumors. Although there are differences in the immunophenotype, clinical history and radiologic examinations are necessary to a definite separation.

SRCLC also may be confused with a wide variety of tumors, including squamous cell carcinoma with clear cell change, clear cells mucoepidermoid carcinoma, balloon cell melanoma, salivary clear cell carcinoma, metastatic clear cell variant of follicular thyroid carcinoma and epithelial-myoepithelial carcinoma.

The clear-cell variant of squamous cell carcinoma also occurs in the sinonasal tract. When the tumor is composed entirely of clear cells, its separation from SRCLC based on hematoxylin and eosin stained sections alone is problematic. The presence of high molecular weight cytokeratin, such as CK5/6, P63, and 34βE12, in the clear cells is the best way to establish the squamous nature of the tumor. Mucoepidermoid carcinoma(MEC) is the most common malignant salivary gland neoplasm, but it also arises in other rare locations, such as the sinonasal tract [14, 15]. Occasionally, clear cells predominate over other cell types, rendering distinction from SRCLC challenging. However, the SRCLC lack the multiple, distinct cell types, such as mucinous cells, squamous cells, and intermediate cells, the latter two of which are more diffusely p63 and CK5/6 positive, that typify EMC. Salivary clear cell carcinoma (CCC), most frequently occurs in intraoral salivary gland sites, e.g., the palate and the base of the tongue but it is rarely found in the nasal cavity [16–18]. CCC is mainly composed of polygonal epithelioid tumor cells arranged into sheets and separated by dense hyalinized stroma, whereas SRCLC can form true glandular, follicular structures, and some neoplastic cells have intranuclear inclusions. In addition, CCC has been shown to harbor WESR1-ATF1 fusion gene [19], which it is easier for distinction between the two tumors. Pure clear-cell variants of melanoma composed predominantly or entirely of cells containing large amounts of clear, vacuolated cytoplasm, due to intracytoplasmic glycogen may be confused with SRCLC. In such cases, immunohistochemical staining is a valuable diagnostic adjunct, as SRCLC is uniformly positive for S100, SOX10 but negative for HMB-45 and Melan-A. Clear cell change may be present in follicular thyroid carcinoma and appears quite vascular, a feature often seen in SRCLC. In such difficult cases, immunostains is helpful in the distinction as thyroid folliculogenic neoplasms are positive for TTF1 and thyroglobulin, both being negative in SRCLC. Epithelial-myoepithelial carcinoma (EMC) consists of a biphasic arrangement of inner luminal ductal cells and outer myoepithelial cells. Some EMC shows a classical tubular growth, with clear, polygonal, abluminal myoepithelial cells and small eosinophilic luminal ducts, the epithelial cells are positive for cytokeratin and EMA. The clear cells stain positive for myoepithelial markers, such as calponin, S-100, and SMA, whereas SRCLC is negative for calponin and SMA.

At present, the histogenesis of SRCLC remains uncertain. New markers for seromucinous glands, including sinonasal glands, namely DOG1, SOX10 and S100 have been defined [20, 21]. DOG1, a calcium-activated chloride channel, initially described in gastrointestinal stromal tumors, but now known to be expressed in normal sinonasal seromucinous glands, it shows moderate apical luminal staining of serous acini with weaker staining in mucous acini. SOX10, an important marker for melanocytic, schwannian and myoepithelial differentiation, is also expressed in normal sinonasal seromucinous glands. S100 is strongly positive in serous acini and weakly positive to negative in the mucous acini. Our study shows that tumor cells in SRCLC are positive for two of these three seromucinous markers, suggesting possible seromucinous derivation of SRCLC. It has also been reported that markers of seromucinous differentiation are present to varying degrees in the majority of low-grade non-intestinal-type adenocarcinomas [21]; a term which has been used synonymously with renal cell -like carcinoma (SRCLC) in the new edition of WHO classification of head and neck tumors [22]. With a common seromucinous phenotype, we conclude that SRCLC is likely a clear cell variant of sinonasal non-intestinal type adenocarcinoma.

SRCLC has a very indolent clinical course. To date, none of the patients with SRCLC have experienced metastatic disease in the neck and distant sites [1, 3, 7, 9, 10], including our case, although the follow up is relatively short. However, owing to its rarity, more cases should be collected to better define short-and long-term clinical behavior, immunohistochemical features, pathogenesis and other relevant information.

## Conclusions

SRCLC is a very rare, low-malignant sinonasal neoplasm. The most striking feature of this tumor is its resemblance to the clear-cell type of renal cell carcinoma. In addition, a panel of sensitive and specific antibodies would also be crucial in making differential diagnosis. Complete resection of this tumor and careful histological examination is necessary to make a correct diagnosis. Early reports of the clinical behavior and natural history suggest that this tumor appears to show very indolent biological behavior with a favorable prognosis.

## Abbreviations

CCC: Salivary clear cell carcinoma
CT: Computer tomography
EMC: Epithelial-myoepithelial carcinoma
RCC: Renal clear cell carcinoma
SRCLC: Sinonasal renal cell-like carcinoma
